# The *Plasmodium vivax* rhoptry neck protein 5 is expressed in the apical pole of *Plasmodium vivax* VCG-1 strain schizonts and binds to human reticulocytes

**DOI:** 10.1186/s12936-015-0619-1

**Published:** 2015-03-07

**Authors:** Gabriela Arévalo-Pinzón, Maritza Bermúdez, Hernando Curtidor, Manuel A Patarroyo

**Affiliations:** Fundación Instituto de Inmunología de Colombia (FIDIC), Carrera 50 # 26-20, Bogotá, Colombia; Universidad del Rosario, Carrera 24 # 63C-69, Bogotá, Colombia

**Keywords:** Malaria, New antigens, *Plasmodium vivax*, *Pv*RON5, Rhoptry neck proteins

## Abstract

**Background:**

Different proteins derived from the membrane or the apical organelles become involved in malarial parasite invasion of host cells. Among these, the rhoptry neck proteins (RONs) interact with a protein component of the micronemes to enable the formation of a strong bond which is crucial for the parasite’s successful invasion. The present study was aimed at identifying and characterizing the RON5 protein in *Plasmodium vivax* and evaluating its ability to bind to reticulocytes.

**Methods:**

Taking the *Plasmodium falciparum* and *Plasmodium knowlesi* RON5 amino acid sequences as template, an in-silico search was made in the *P. vivax* genome for identifying the orthologous gene. Different molecular tools were used for experimentally ascertaining *pvron5* gene presence and transcription in *P. vivax* VCG-1 strain schizonts. Polyclonal antibodies against *Pv*RON5 peptides were used for evaluating protein expression (by Western blot) and sub-cellular localization (by immunofluorescence). A 33 kDa *Pv*RON5 fragment was expressed in *Escherichia coli* and used for evaluating the reactivity of sera from patients infected by *P. vivax*. Two assays were made for determining the RON5 recombinant fragment’s ability to bind to reticulocyte-enriched human umbilical cord samples.

**Results:**

The *pvron5* gene (3,477 bp) was transcribed in VCG-1 strain schizonts and encoded a ~133 kDa protein which was expressed in the rhoptry neck of VCG-1 strain late schizonts, together with *Pv*RON2 and *Pv*RON4. Polyclonal sera against *Pv*RON5 peptides specifically detected ~85 and ~30 kDa fragments in parasite lysate, thereby suggesting proteolytic processing in this protein. Comparative analysis of VCG-1 strain *Pv*RON5 with other *P. vivax* strains having different geographic localizations suggested its low polymorphism regarding other malarial antigens. A recombinant fragment of the *Pv*RON5 protein (r*Pv*RON5) was recognized by sera from *P. vivax*-infected patients and bound to red blood cells, having a marked preference for human reticulocytes.

**Conclusions:**

The *pvron5* gene is transcribed in the VCG-1 strain, the encoded protein is expressed at the parasite’s apical pole and might be participating in merozoite invasion of host cells, taking into account its marked binding preference for human reticulocytes.

**Electronic supplementary material:**

The online version of this article (doi:10.1186/s12936-015-0619-1) contains supplementary material, which is available to authorized users.

## Background

The rhoptries, together with the micronemes, form the main secretory organelles of most infective forms of parasites from *Apicomplexa* (the phylum to which *Plasmodium* belongs) [1]. The importance of the rhoptries is reflected in the wide range of proteins contained in these organelles, which are involved in invasion of host cells. Some of these proteins are restricted to the apical duct (known as the rhoptry neck) or to the rhoptry bulb, which is characterized by having a high lipid content [2]. Protein spatial localization within the rhoptries in malaria allows the parasite to carry out different functions during the coordinated invasion of its host cell, which has been correlated with each protein’s release time [3]. Proteins from the rhoptries are thus implicated in specific recognition of the host cell, in tight or moving junction (TJ-MJ) formation, parasitophorous vacuole formation and host cell remodelling [3,4].

Rhoptry neck proteins (called RONs) have been strongly associated with the formation of the TJ, an electron dense circular structure which is formed between the parasite and the host cell, constituting the central axis where the different invasion events become organized [5]. RON2, RON4 and RON5 proteins have been identified in the TJ formed by *Plasmodium falciparum* (and RON8 in *Toxoplasma gondii*), associated with a micronemal protein called apical membrane antigen 1 (AMA-1) [6-8]. Besteiro *et al.,* described an organizational model of MJ in *T. gondii* for the first time, consisting of a multi-protein rhoptry/microneme complex where it has been suggested that the parasite supplies its own receptors (RON proteins) for gaining access to the host cell [9]. The different interactions between MJ components have been mapped in detail since these first studies. Crystallization studies of the AMA-1 ectodomain in complex with a RON2 extracellular peptide have revealed a conformational change in AMA-1 domain II leading to a perfect fit having high affinity between both proteins [10,11]. The description of this interaction has provided the molecular basis for understanding the invasion inhibition mechanisms displayed by the 4G2 [12] and 1F9 [13] monoclonal antibodies directed against *Pf*AMA-1 and peptides identified from random peptide libraries expressed on phage surface, such as R1 [14]. *PfR*ON2 binding to *Pf*AMA-1 is not affected by *Pf*AMA1’s high polymorphism in the parasite’s distinct strains; furthermore, the *Pf*RON2 peptide which binds to the hydrophobic groove shows crossed invasion inhibition between strains [11], thereby highlighting its biological importance in developing prophylactic methods. Interestingly, a recent study has shown that immunization with the AMA1-RON2 functional complex, but not with individual antigens, induced complete antibody-mediated protection against homologous experimental challenge with the lethal *Plasmodium yoelii* YM strain [15]. Such protection would seem to be partly mediated by antibodies having specificity for new epitopes surrounding the RON2 binding site [15].

While different studies have established the importance of RON2-AMA-1 interaction, other ones obtaining partial or total AMA-1 knockouts, have led to questioning the role of this protein in the TJ formation [16,17]. It has been shown that the absence of AMA-1 in *T. gondii* is complemented and/or compensated by two homologous genes [18]; however, RON proteins continue to gain importance in spite of such discrepancies. Bearing in mind that it has not been possible to inactivate the *ron4* gene in *Plasmodium berghei*, following various attempts to do so, it has been suggested that the RON4 protein plays an important role in merozoites [17]. A significant reduction in the invasion of hepatic cells has been found following conditional silencing of the *ron4* gene in *P. berghei* sporozoites [17]. It has been found to date that the *T. gondii* RON4 protein carboxyl terminal region, but not that of *P. falciparum,* has been associated with the tubulin β-chain in mammalian cells, thereby suggesting RON4 translocation to host cell cytoskeleton, acting as anchoring site for parasite entry [19] and partly confirming the aforementioned model proposed by Besteiro *et al.* [9].

Few studies have dealt with a functional role for *Pf*RON5 within the TJ, even though some methodological approaches have shown its association with the RON/AMA1 multi-protein complex [9,12]. It has been shown recently that the conditioned absence of RON5 in tachyzoites caused the complete degradation of *Tg*RON2 and incorrect *Tg*RON4 localization in the rhoptries, having significant implications within the parasite’s invasion cycle [20]. The functional dissection of each *Tg*RON5 region has led to it becoming established that the *Tg*RON5 prodomain, together with the last portion of the *Tg*RON5 amino region (RON5N), participate in the correct targeting of the rhoptry neck proteins, while the carboxyl terminal region is essential for stabilizing *Tg*RON2 [20]. Interestingly, it has been described that the *P. falciparum Pf*RON5 sequence contains peptides, which bind saturably, and having high affinity for binding to receptors on red blood cell (RBC) membrane which are sensitive to enzyme treatment [21]. Such peptides, called high activity binding peptides (HABPs), can inhibit merozoite *in vitro* invasion of their host cells [21], thereby highlighting the role of *Pf*RON5 during parasite invasion. Although most studies have described the possible translocation of some RONs to the host cell membrane, its mechanisms and signals have not been explored in detail. This translocation could be preceded by RON proteins’ specific interaction with receptors on RBC membrane, as has been previously reported for *Pf*RON5 [21] and *Pf*RON2 [22]. Such interaction might have functional relevance when designing control methods aimed at blocking RON-AMA1 complex formation.

Taking into account the major role that RON proteins display in *T. gondii* and *P. falciparum* parasite invasion cycles, a comparative approach together with an adaptation of a *Plasmodium vivax* strain in *Aotus* monkeys [23] has been used for identifying and characterizing new proteins, such as *Pv*RON2 [24] and *Pv*RON4 proteins [25] in the second-most important species causing malaria around the world: *P. vivax* [26]. Such approach has provided the basis for advances made in identifying *P. vivax* proteins containing important characteristics which are typical of vaccine candidates, such as expression in late schizonts, localization on/in cell membrane or secretory organelles and, in some cases, high antigenic and immunogenic capacity [27]. Some antigens, such as merozoite surface protein-1 (*Pv*MSP-1), reticulocyte binding protein-1 (*Pv*RBP-1) and the Duffy binding protein (*Pv*DBP), have reticulocyte-specific binding sequences and have been considered to date among the most promising vaccine candidate antigens [27].

The gene encoding the *Pv*RON5 protein was identified in the present work; it consisted of a 1,158 residue antigen which was found to be conserved among *P. vivax* strains from different geographical regions and which was found to be expressed at *P. vivax* schizonts’ apical pole, together with *Pv*RON4 and *Pv*RON2 proteins. *Pv*RON5 anti-peptide antibodies recognized two bands in *P. vivax* schizont lysate, suggesting that proteolytic processing could be implicated in the protein’s functional activation. Analytical and molecular biology techniques led to obtaining the r*Pv*RON5, which was recognized by sera from patients having active *P. vivax* infection. Immunoprecipitation and immunofluorescence studies have shown that r*Pv*RON5 binds to reticulocyte-enriched samples, suggesting that this protein might be involved in merozoite invasion of human reticulocytes.

## Methods

### In-silico search for *pfron5* and *pkron5* homologous gene in *Plasmodium vivax*

The presence of the *pvron5* gene in the *P. vivax* genome was evaluated by using the *P. falciparum* (PF3D7_0817700) and *Plasmodium knowlesi* (PKH_051420) RON5 protein amino acid (aa) sequences deposited in PlasmoDB [28] and the Basic Local Alignment Search Tool (BLAST) [29], from the National Center for Biotechnology Information (NCBI) as template. Genscan [30], Spidey [31] and tBlastn were used for determining the presence and boundaries of the exons and introns present in the *pvron5* gene. The study of synteny considered the presence of open reading frames (ORFs), gene structure, transcription direction, and values regarding identity and similarity between *P. vivax*-*P. knowlesi* and *P. vivax*-*P. falciparum* species.

Primers were designed on the ORF having the highest score in bioinformatics’ analysis based on the results for the above. Three sets of primers were designed for the complete amplification of genomic DNA (gDNA) while two primers were designed for complementary DNA (cDNA) covering from the start codon to the stop codon. Different primers were needed for the complete sequencing of cDNA. Table 1 lists the forward and reverse primers used for PCR amplification and sequencing for each amplicon.

**Table 1:** **Primers used in amplifying and sequencing** ***Pv*** **RON5 from gDNA and cDNA**

**Name**	**Sequence 5 → 3**	**Amplicon**	**Target**
A1	CGT CTG TAA GAC CTC CC	3.404 bp	gDNA
A3	CGA TGA AGC CCT TCT CC		gDNA
A5	CGG GAC AAG CTG AAT AAC	1.815 bp	gDNA
A7	TGA CGT CGG CGC AGA TG		gDNA
A9	AGT GCC TCC ATG GAC AAT A	3.158 bp	gDNA
A11	GCT GAT CGG TCG GCT GA		gDNA
*Pv*RON5-F	ATG CTG AAG TAC GTG CTA CTC	3.477 bp	cDNA
*Pv*RON5-R	GGG TAT CCT CGT GTG CAC		cDNA
*Pv*RON5-sec-F1	CGT CTG TAA GAC CTC CC	NA	cDNA sequencing
*Pv*RON5-rev-R1	CGA TGA AGC CCT TCT CC	NA	cDNA sequencing
*Pv*RON5-sec-F2	CGG GAC AAG CTG AAT AAC	NA	cDNA sequencing
*Pv*RON5-sec-R2	TGA CGT CGG CGC AGA TG	NA	cDNA sequencing
*Pv*RON5-sec-F3	AGT GCC TCC ATG GAC AAT A	NA	cDNA sequencing
*Pv*RON5-sec-R3	GCT GAT CGG TCG GCT GA	NA	cDNA sequencing

NA: Not applicable, gDNA: genomic deoxyribonucleic acid, cDNA: complementary deoxyribonucleic acid.

Different bioinformatics tools were used for evaluating important motifs and domains in the hypothetical *Pv*RON5 sequence. Signal P software was used for evaluating signal peptide presence [32], Polyphobius for determining the presence of transmembrane domains [33] and PredGPI for GPI anchors [33]. The BaCelLo tool was used for predicting *Pv*RON5 sub-cellular localization [34]. Sequence tandem repeat extraction and architecture modelling (XSTREAM, variable ‘X’) was used for finding repeat sequences and the simple modular architecture research tool (SMART) for searching for other important motifs and domains [35].

### The source of nucleic acids and cDNA synthesis

Vivax Colombia Guaviare 1 (VCG-1) strain parasites were used as source for DNA, RNA and parasite proteins. These samples had been obtained as described previously [23] and after nucleic acid and protein extraction, stored at −70°C until use. RNA extracted by using the TRIzol method [36] and Superscript III enzyme (Invitrogen), were used for cDNA synthesis following the manufacturer’s specifications.

### PCR conditions, cloning and sequencing

KAPA HiFi HotStart DNA polymerase (Kapa Biosystems) was used for amplifying *pvron5* from gDNA in 25 μL final reaction volume containing 12.5 μL 2x KAPA HiFi Ready Mix, 1.5 μL of each primer (Table 1) at 5 μM concentration and 7.5 μL of nuclease-free water. The amplification conditions for the three products amplifying gDNA consisted of one 3-min cycle at 95°C followed by 35 cycles lasting 20 sec at 98°C, 15 sec at 60°C and 3 min 30 sec at 72°C with a final extension cycle lasting 5 min at 72°C. cDNA was amplified with *Pv*RON5-F and *Pv*RON5-R primers and the TAQXpedite high fidelity enzyme (Epicentre Biotechnologies) at final 25 μL volume, according to the manufacturer’s recommendations. A denaturing cycle was used which lasted 30 sec at 95°C followed by 35 cycles at 95°C for 10 sec, 58°C for 10 sec and 72°C for 2 min. The same conditions and previously described primers were used for amplifying the *pvrhoph3* gene from cDNA [37].

The amplified products were visualized on 1% agarose gels and their molecular weights calculated based on a molecular weight marker. The product obtained from cDNA was purified by using an Ultra Clean Gel Spin DNA purification kit (MOBIO Laboratories), according to the manufacturer’s specifications. An additional reaction was needed for adding adenines to the product’s 3′ end for its TA cloning into pGEM-T vector (Promega). The construct was used for transforming TOP10 cells and three positive clones were selected which had been obtained from independent PCR reactions which were sent for sequencing on an ABI PRISM 310 Genetic Analyzer. CLC DNA Workbench (CLC bio) software was used for analyzing the sequences; the resulting cDNA sequence was deposited in GenBank. Clustal W software was used for comparing the VCG-1 strain’s cDNA sequence to those of the Sal-1, Mauritania I, Brazil I, North Korean and India VII strain sequences [38].

### *Pv*RON5 recombinant fragment cloning, expression and purification

A nucleotide construct encoding 296 aa from *Pv*RON5 (residues 863T to 1158P) was commercially synthesized by GenScript, thereby optimizing a variety of parameters (i.e. *Escherichia coli* codon bias, GC content and mRNA secondary structure), which are critical for the gene fragment’s efficient expression. The synthetic gene was ligated in pQE30 vector (Qiagen) BamHI and HindIII sites and the new construct was named pQE30-rRON5. This plasmid was used for transforming JM109 strain *E. coli* cells, followed by sequencing with the vector’s primers.

Kanamycin-resistant and ampicillin-sensitive M15 cells were transformed with the pQE30-rRON5 plasmid. r*Pv*RON5 expression was obtained by inoculating 200 mL Luria Bertani (LB) medium containing 0.1 mg/mL ampicillin and 25 μg/mL kanamycin with 10 mL culture grown overnight. The culture was grown in conditions involving constant shaking at 37°C until 0.4-0.6 optical density was reached at 620 nm. r*Pv*RON5 expression was induced by adding isopropyl β-D-1-thiogalactopyranoside (IPTG, Invitrogen) at 1 mM final concentration for 4 hr at 37°C with constant shaking. The cell culture was spun at 10,000 × g for 30 min at 4°C and the bacterial pellet containing the protein in inclusion bodies was washed twice with buffer A (20 mM Tris–HCl pH 8.0, 1 mM EDTA, 1 mM iodoacetamide, 1 mM PMSF and 1 μg/mL leupeptin) supplemented with lysozyme, followed by cell disruption using a sonicator (Branson). Following the aforementioned treatment, the inclusion bodies were recovered by spinning at 10,000 × g for 30 min at 4°C and washed once with buffer A supplemented with 2 M urea. The recombinant protein was then solubilized with lysis buffer containing 10 mM Tris–HCl, 100 mM NaH_2_PO_4_, 6 M urea, 10 mM imidazole and 10% glycerol. Protein presence in the supernatant was evaluated by Western blot using an anti-polyhistidine monoclonal antibody (Sigma).

The recombinant protein (r*Pv*RON5) was purified by affinity chromatography using Ni^2+^-NTA agarose (Qiagen), as recommended by the manufacturer. Briefly, the supernatant was placed on a Ni^2+^-NTA column, which had been previously equilibrated in lysis buffer. The column was extensively washed with lysis buffer and then washed with decreasing amounts of urea (6–0 M) in the same lysis buffer. The recombinant protein was then eluted with an imidazole linear gradient (0.03 M-0.5 M) in washing buffer (10 mM Tris–HCl, 100 mM NaH_2_PO_4_ at pH 8.0). The fractions were analyzed using SDS-PAGE and stained with Coomassie blue. The fractions containing a single band at the expected height were pooled and dialyzed against 1X phosphate buffer. Concentration was determined by the bicinchoninic acid method (Bio-Rad) using bovine serum albumin (BSA, Sigma) as standard.

### Producing polyclonal antibodies against *Pv*RON5, *Pv*RON4 and *Pv*RON2 proteins

The hypothetical *Pv*RON5 sequence was used for predicting linear B-cell epitopes. Selected peptides were synthesized for rabbit immunization and producing polyclonal antibodies, as has been described previously [24]. Peptides were chemically synthesized in solid phase and one cysteine and one glycine (CG) were added to the amino and carboxy termini during synthesis. Each peptide was lyophilized and analyzed by reverse phase high performance liquid chromatography (RP-HPLC) and MALDI-TOF mass spectrometry (Auoflex, Bruker Daltonics). Polymer peptides 36927 (CG^775^ATR*T*DHFSRSASMDNNKKSR^794^GC) in which a cysteine (C) had been replaced by threonine (T) in position 778, 36930 (CG^351^NASYDLEEYQNEFKPTNTSQ^370^GC) and 39274 (CG^69^MFDPKDKKFVPSKSKKAHIV^88^GC) were selected for antibody production. Antibodies thus obtained were later used for evaluating protein expression in late schizonts. Synthetic peptide 39276 (CG^989^GIDEDNERFYVLQDKTKVPE^1008^GC) was inoculated for producing antibodies recognizing r*Pv*RON5 in binding assays.

Four New Zealand rabbits (numbered 2, 3, 32, and 60) were then selected after having been evaluated by Western blot as being negative for recognition of *P. vivax* proteins. Rabbits 2 and 3 were subcutaneously immunized with 500 μg of peptides 39274 and 39276 respectively, emulsified in Freund’s complete adjuvant (FCA), while rabbits 32 and 60 were inoculated with a mixture of peptides 36927 (250 μg) and 36930 (250 μg) in FCA. Booster immunizations on days 20 and 40 were administered using the same peptides emulsified in Freund’s incomplete adjuvant (FIA). Antibodies directed against *Pv*RON4 and *Pv*RON2 proteins were used for co-localization studies, inoculating the previously described peptides in mice [24,25]. This involved taking two BAlb/C mice which were intraperitoneally immunized (ip) with 75 μg *Pv*RON2 polymer peptides 35519 and 35520 [24] emulsified in FCA, while another two mice were immunized with *Pv*RON4 peptides 36114 and 36115 [25]. Three boosters were given on days 30, 45 and 60 using the same peptides emulsified in FIA. Rabbit and mouse sera was collected on day 60 and 75, respectively, and used for further assays. Animal immunization and bleeding was done according to Colombian animal protection recommendations (Law 84/1989 and Resolution 8430/1993) for handling live animals for research or experimentation purposes. All experimental procedures involving animals had been previously approved by the Fundación Instituto de Inmunología’s ethics committee.

### *Pv*RON5 polymer peptide recognition by rabbit sera

Polymer peptides (1 μg) were sown in 96-well ELISA plates, as previously described [39]. The plates were incubated with sera from each rabbit in 1:100 dilution for 1:30 min at 37°C, followed by three washes with 0.05% PBS-Tween. Peroxidase-coupled anti-rabbit (Vector Laboratories) at 1:5,000 dilution was used as secondary antibody. Immunoreactivity was revealed by using a TMB Micro-well Peroxidase Substrate System kit (KPL Laboratories), according to the manufacturer’s instructions. Absorbance was read at 620 nm on an ELISA reader (Lab Systems Multiskan MS).

### Evaluating *Pv*RON5 expression in late schizonts

*Pv*RON5 expression was determined by Western blot (WB) and immunofluorescence. Regarding WB, the parasite proteins extracted from the VCG-1 strain were electrophoresed in reducing conditions on 10% polyacrylamide/SDS gels (SDS-PAGE). The proteins were then transferred to polyvinylidene difluoride (PVDF) membranes, which had been previously activated with methanol. Following transfer, the membrane containing the parasite proteins was blocked with a 5% milk solution in 0.05% PBS-Tween and 3-mm wide strips were cut to be incubated with the sera (pre-immune or immune) from rabbits immunized with *Pv*RON5 polymer peptides (1:40 dilution in blocking solution). In another assay, immune serum was pre-incubated with inoculated polymer peptides before being incubated with the PVDF membrane. Each strip was washed thrice with 0.05% PBS-Tween and incubated with anti-rabbit phosphatase-coupled antibodies (Biomedicals) in 1:5,000 dilution. The reaction was revealed by using a BCIP/NBT kit (Promega), according to the manufacturer’s instructions.

Indirect immunofluorescence (IFA) involved using a previously described protocol, with some modifications [25]. Briefly, the eight-well chamber slides containing parasitized RBCs were fixed with 4% formaldehyde and then permeabilized for 10 min with 1% v/v Triton X-100. The slides were blocked with 1% PBS/BSA solution at 37°C for 30 min and incubated with primary anti-*Pv*RON5, *Pv*RON2 or *Pv*RON4 antibodies in 1:40 dilution in blocking solution. Fluorescein-labelled anti-rabbit IgG (FITC) (Vector Laboratories) and rhodamine-labelled anti-mouse IgG (Millipore) were used as secondary antibodies. Parasite nuclei were stained with 4′, 6-diamidino-2-phenylindole (DAPI) and fluorescence was visualized by fluorescence microscope (Olympus BX51) using an Olympus DP2 camera and Volocity software (Perkin Elmer).

### Antigenicity studies with r*Pv*RON5

The r*Pv*RON5 recombinant protein was electrophoresed on 12% SDS-PAGE gels and then transferred to PVDF membranes. The membrane was blocked and cut into 3-mm wide strips which were incubated with sera from *P. vivax*-infected patients (1:100) who were living in different endemic regions in Colombia. Healthy individuals’ sera were used as controls. The membranes were then incubated with phosphatase-coupled anti-human antibodies (Biomedicals) in 1:4,000 dilution and the reaction was revealed with a BCIP/NBT kit, according to the manufacturer’s instructions.

The r*Pv*RON5 recognition was determined by ELISA. In brief, 96-well plates coated with 2 μg r*Pv*RON5 were incubated with a 1:100 dilution of serum samples from *P. vivax*-infected individuals and 1:5,000 anti-human IgG as secondary antibody.

### Obtaining reticulocyte-enriched fractions

A previously described protocol was followed for reticulocyte enrichment [40], with some modifications. Around 30 ml of umbilical cord blood (n = 5) were obtained from the District Blood Bank in Bogotá, Colombia. The plasma was removed after being washed in PBS several times. The packed RBC were diluted ten times in PBS and passed three times through CF11 columns (Whatman) to eliminate platelets and white cells. A 70% Percoll solution in 0.15 M NaCl was prepared from a Percoll stock in 9:1 Percoll-1.5 M NaCl ratio. The RBC were spun, diluted by half and placed on the 70% Percoll gradient which was spun at 2,500 × *g* for 25 min. The fine reticulocyte band formed on the Percoll interface was carefully removed and then washed twice with PBS. Reticulocyte viability and count were monitored in each process using cresyl blue supravital stain.

### r*Pv*RON5 binding to reticulocyte-enriched samples

The interaction between r*Pv*RON5 with reticulocyte-enriched samples and RBC passed through CF11 columns was determined in two experiments. The first involved incubating 100 μL r*Pv*RON5 recombinant protein with 100 μL RBC or enriched reticulocytes, taken to 300 μL final volume with PBS at 4°C overnight. The mixture was passed through a 400 μL dibutyl phthalate cushion and spun at 2,500 × *g* for 5 min. The supernatant was skimmed off and the pellet was washed twice with PBS before eluting the protein with PBS/1 M NaCl. The supernatant was pre-incubated at 4°C for 3 hr with protein G conjugated sepharose beads (GammaBind Plus Sepharose, GE Healthcare) diluted to 50% in NETT buffer (50 mM Tris–HCl, 0.15 M NaCl, 1 mM EDTA, and 0.5% Triton X-100) supplemented with 0.5% BSA. The recovered supernatants were incubated with anti-polyhistidine monoclonal antibody (Sigma) in 1:4,000 dilution with gentle shaking at 4°C for 5 hr and 20 μL 50% protein G-conjugated beads were then added. Following incubation, the mixture was spun at 3,800 × *g* for 5 min and the beads were washed once with NETT-0.5% BSA. The immune-precipitated recombinant protein was extracted from the beads by incubation with SDS-PAGE reducing loading buffer at 100°C for 3 min. Supernatants were collected for WB analysis using peroxidase-coupled anti-histidine monoclonal antibody.

The second assay involved 5% reticulocyte-enriched samples in HEPES buffered saline (HBS) solution being incubated with 15 μg r*Pv*RON5 at 4°C overnight. The samples were spun, washed once with HBS buffer and 50 μL bis (sulfosuccinimidyl) suberate (BS^3^-Pierce) was added at 250 μg/mL final concentration for 1 hr at room temperature (RT) with slow shaking. The samples were then washed twice with HBS buffer and the RBC or reticulocytes were blocked with 1% BSA in HBS buffer for 1 hr at 4°C. Following two washes with HBS, the samples were incubated with primary anti-RON5 antibody (rabbit 3) in 1:40 dilution and anti-CD71 monoclonal antibody (Life Technologies) in 1:100 dilution for 1 hr at 4°C. Antibody which did not bind was removed by two washings with HBS. The samples were then incubated with fluorescein-labelled anti-rabbit IgG (FITC) (Vector Laboratories) and rhodamine-labelled anti-mouse IgG (Millipore). Associated fluorescence was visualized on a fluorescence microscope (Olympus BX51) using an Olympus DP2 camera and Volocity software (Perkin Elmer). The following negative controls were included for evaluating non-specific reactivity between polyclonal antibody (recognizing r*Pv*RON5) and secondary antibody with proteins on target cell membrane: reticulocytes or RBC incubated only with polyclonal antibody against r*Pv*RON5 followed by FITC-coupled secondary antibodies, reticulocytes or RBC incubated with only FITC-coupled secondary antibodies and reticulocytes or RBC incubated with r*Pv*RON5 followed by incubation with FITC-coupled secondary antibody.

### Statistical analysis

Differences in antibody production between pre-immune and post-third immunization rabbit sera obtained by ELISA were evaluated using non-parametric Wilcoxon signed rank test. Ten photos were selected for evaluating the differences between reticulocyte-associated fluorescence compared to RBC-associated in r*Pv*RON5 target cell binding assays; in each photograph, reticulocytes and RBCs were numbered. One reticulocyte and one RBC were randomly chosen in each photo for measuring 256 fluorescence points per cell using ImageJ 1.48 software. The 512 data obtained per photo were loaded into the SPSS software (version 20.0) and the differences evaluated by non-parametric Wilcoxon signed rank test. The images presented in the manuscript are representative of at least 10 individual observations.

## Results

### In-silico identification and molecular characterization of the *pvron5* gene

In spite of a lack of an *in vitro P. vivax* continuous culture providing sufficient parasite samples for study, a comparative approach involving other important species such as *P. falciparum* and the use of *Aotus* monkeys for maintaining a *P. vivax*-adapted strain [23] has led to the identification of a significant number of antigens (16 new proteins) which could be participating in invasion [27].

**Figure 1:**
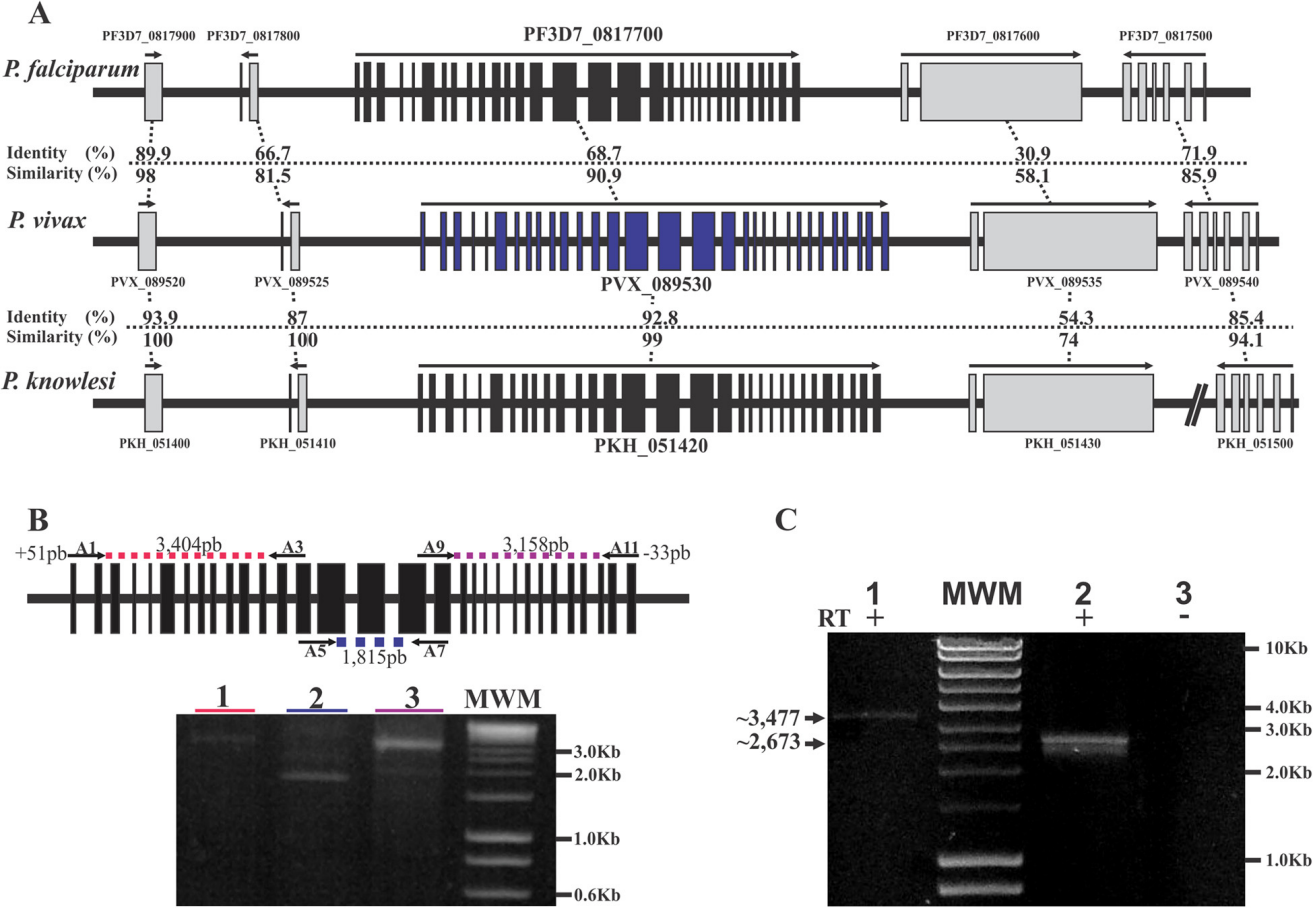
**In-silico, genomic and transcriptional analysis of the** ***pvron5*** **gene in the** ***Plasmodium vivax*** **VCG-1 strain. (A)** Schematic representation of the *pvron5* gene (blue) localized in Pv_Sal1_chr05:629000..649500 (21 kb) and comparative analysis with *P. falciparum*: Pf3D7_08_v3:798000..818000 (20 kb) and *P. knowlesi*: Pk_strainH_chr05:663000..682000 (19 kb) and Pk_strainH_chr05:709,700..711,400 (1.7 kB). The transcription direction is shown for each gene and the identity and similarity values between *ron5* and adjacent genes regarding the three *Plasmodium* species. Analysis was made concerning the information available in PlasmoDB [28] **(B)** *pvron5* amplification from gDNA. Above, a representation of the *pvron5* gene and the localization of the three sets of primers used for amplifying the gene are shown, along with the size of the expected products. ~3,404 bp (lane 1), ~1,815 bp (lane 2) and ~3,158 bp (lane 3) bands can be observed on the agarose gel, showing the weight of the expected products for *pvron5*. MWM: molecular weight marker. **(C)** *pvron5* transcription in VCG-1 strain late schizonts. Lane 1: *pvron5* positive RT-PCR. Lane 2: *pvrhoph3* positive RT-PCR. Lane 3: *pvron5* negative RT-PCR.

The gene encoding the *Pv*RON5 protein was identified in this study; this antigen is expressed in the schizont phase of the intra-erythrocyte cycle. An initial search was made in tBlastn regarding the *pvron5* gene using the *Pf*RON5 aa sequence (1,156 aa). The analysis revealed a high probability that the target gene would be localized in chromosome 5 (Pv_sal1_chr05) of the *P. vivax* genome between base pairs 635,954 to 642,743 (i.e., *Pf*RON5 aa 105 to 1,156). The *P. knowlesi* RON5 aa sequence was used for searching for the start codon, as this parasite was phylogenetically closest to *P. vivax*. This analysis revealed that the *pvron5* gene’s structural region began in position 634,671 and ended in chromosome 5 nucleotide 642,743; a gene (PVX_089530) having a putative function was found in this position (Figure 1A). Such analysis has been indispensable in re-annotating genes such as *pvron4* [25] and *pvrhoph3* [37] where incorrect annotations have been found regarding the start and termination of the gene as well as the number of exons.

It was also found that transcription direction, *pvron5* gene structure regarding *pfron5* and *pkron5* and the presence of 31 exons in each species analyzed was conserved (Figure 1A). *ron5* gene identity (ID) and similarity (SI) values between the three species were above 60%, ID and SI being greater between *P. vivax*-*P. knowlesi* (92% ID; 99% SI) regarding *P. vivax*-*P. falciparum* (68% ID; 90% SI); such values agreed with the evolutionary relationships between these parasites. Gene structure, transcription orientation and SI values above 35% were taken into account for determining the existence of conserved synteny in the chromosome region where *pvron5* was localized [41]. The results revealed conservation regarding the number of introns and exons of the genes upstream and downstream *ron5* as well as SI values extending up to 100% in some genes (Figure 1A), thereby suggesting that the *pvron5* gene is an orthologue of the *pfron5* and *pkron5* genes.

Following bioinformatics analysis, the presence of the *pvron5* gene in the VCG-1 strain genome was investigated by PCR amplification of ~8,076 bp comprising the gene from gDNA (Figure 1B); ~3,404 bp, ~1,815 bp and ~3,158 bp fragments were amplified with three sets of overlapping primers (by about 100 bp), two of which were designed upstream (+51) and downstream (−33) of the gene of interest (Figure 1B).

### *pvron5* gene transcription in the late phase intra-erythrocyte cycle

Merozoite invasion of RBC is a complex process, during which the parasite recognizes its target cell, orientates its apical pole and becomes internalized through the parasite’s machinery and RBC membrane invagination [42]. These invasion events are accompanied by a large number of receptor-ligand interactions between proteins localized on/in the membrane or the parasite’s apical organelles with RBC membrane proteins [42]. *Plasmodium falciparum* transcriptome studies have shown that ~500 ORFs have a transcription peak in late-schizont stage and that 60 to 90 antigens could be participating in invasion [43]. The *pfron5* gene has a maximum transcription peak after 41 hr of the intra-erythrocyte cycle, this being an appropriate time for its participation in the invasion cycle. In fact, it has been reported that *Pf*RON5 is associated with important antigens involved in invasion, such as RON2 and AMA1 [6,8].

PCR amplification of a product having around 3,477 bp from cDNA synthesized with reverse transcriptase (RT+) confirmed *pvron5* gene transcription (Figure 1C). The *pvrhoph3* gene was amplified as transcription control during schizont stage. Amplification was not obtained from RT negative samples, showing that synthesized cDNA was free of contamination with gDNA (Figure 1C). *pvron5* transcription in the VCG-1 strain agreed with previous studies of the *P. vivax* transcriptome carried out with three clinical isolates, showing that the PVX_089530 gene had a maximum transcription peak between 35 (TP7) and 40 hr (TP8) of the intra-erythrocyte cycle, similar to that reported for proteins which have been well characterized regarding invasion, such as *Pv*200, or new antigens, such as *Pv*RON2 [44]. Large differences regarding the size of the amplified *pvron5* product from gDNA (~8,076 bp) and cDNA (3,477 bp) suggested the presence of intron regions, this being consistent with the bioinformatics prediction (Figure 1).

**Figure 2:**
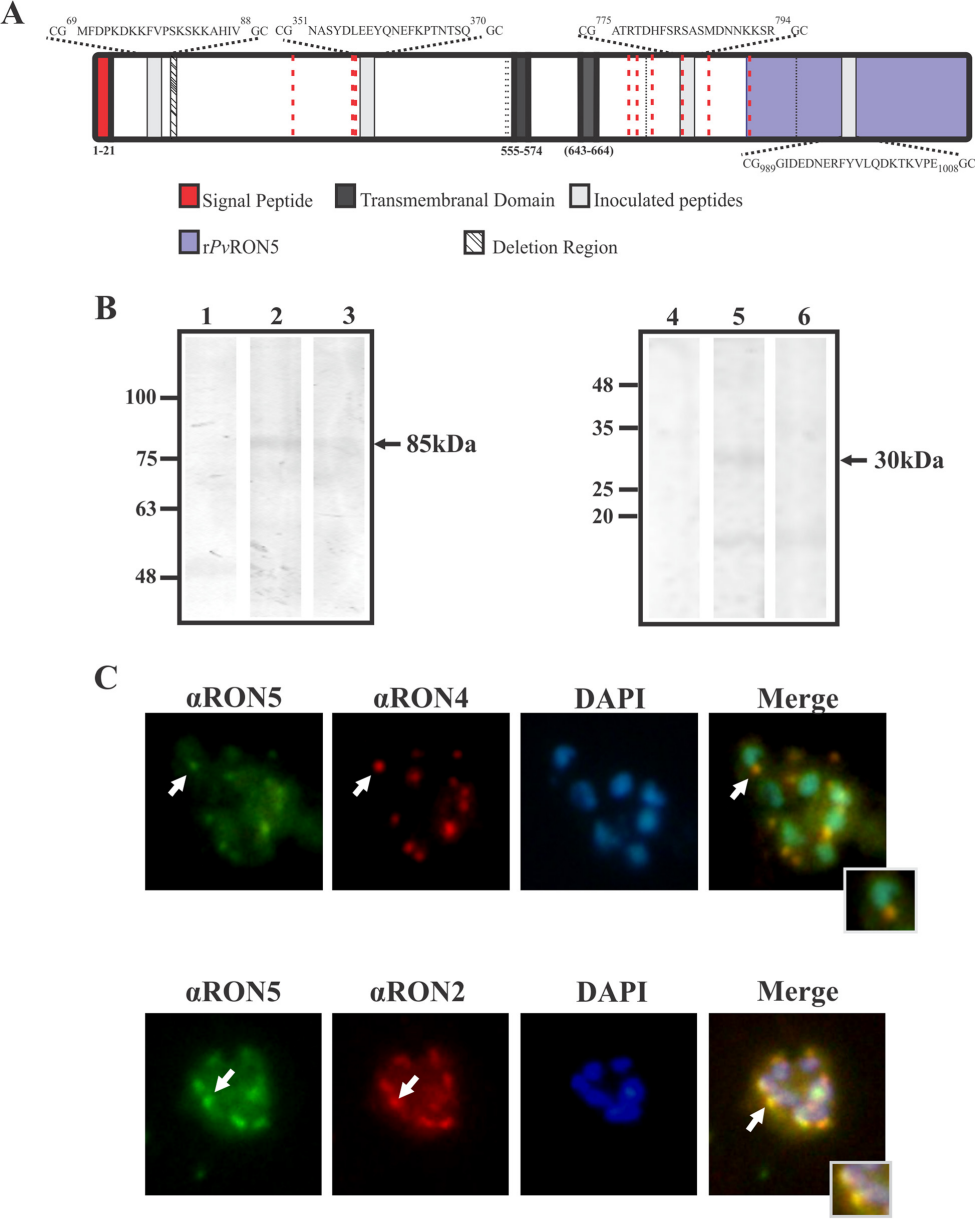
***Pv*** **RON5 expression in** ***Plasmodium vivax*** **schizonts. (A)** Representation to scale of the *P. vivax* RON5 protein. The signal peptide and the two transmembrane domains predicted by bioinformatics tools and the localization and sequence of the linear B-cell epitope peptides selected for polyclonal antibody production are shown. Comparative analysis of amino acid sequences from different *P. vivax* strains revealed the deletion of a nine-residue-long region; black dotted lines show synonymous and non-synonymous changes. Aligning *P. vivax*, *P. falciparum* and *P. knowlesi* RON5 revealed nine conserved cysteines (red dotted lines). The recombinant protein (r*Pv*RON5) produced in *E. coli* is shown in purple*.* **(B)** *Pv*RON5 expression in VCG-1 strain schizont lysate. Lane 1, pre-immune serum 60; lane 2, immune serum 60; lane 3, immune serum 60 pre-incubated with peptides 36930 and 36927; lane 4, pre-immune serum 2; lane 5, immune serum 2 and lane 6, immune serum 2 pre-incubated with peptide 39274. **(C)** *Pv*RON5 sub-cellular localization in *P. vivax*-infected RBCs in schizont stage. Green shows serum reactivity for *Pv*RON5, having a dotted pattern similar to that observed for *Pv*RON4 and *Pv*RON2 (red). The arrows show the dotted pattern and the overlaying of the images (merging). DAPI (4′,6-diamidino-2-phenylindole) was used for staining the parasite nucleus.

The *pvron5* cDNA sequence (VCG-1 strain) was deposited in the NCBI (GenBank accession number: KP026121) and was compared to strains distributed throughout different geographic regions, such as India, Brazil, Asia and Africa, taking the Sal-1 strain deposited in PlasmoDB as reference. Analysis at nucleotide level revealed a 27 nt deletion towards the 5′ extreme in the India VII strain, coinciding with the absence of nine amino acids between positions 100 to 108 (Figure 2A and Additional file 1). Six changes were also found, four of which were non-synonymous mutations in positions 544, 547, 730, and 929 at amino acid level (Figure 2A). Such amount of change in *Pv*RON5 is low when compared to that present in other malarial antigens, such as MSP-1 and AMA-1 [45,46]. In fact, amino acids conserved physical-chemical properties in the first three changes mentioned (544, 547 and 730). However, it cannot be ruled out that such changes could have been associated with parasite evasion mechanisms. This is important when designing a completely effective anti-malaria vaccine as antigens having high genetic variability involve the expression of different alleles in different parasite strains, inducing allele-specific responses partly reducing vaccine efficacy [47,48].

### Analyzing RON5 expression and localization in *Plasmodium vivax*

RON5 is a 1,158 residue-long protein in the *P. vivax* VCG-1 strain and, according to SignalP and BaCelLo, it is a secreted protein containing a classical eukaryotic signal sequence towards the amino terminal extreme in the first 21 amino acids, having a short potential site for a type I peptidase between aa 21(S) and 22(R) (Figure 2A). This allows entry to the secretory pathway for traffic from the endoplasmic reticule through the Golgi complex by a conserved pathway before being packaged into the apically located secretory organelles. It also contains two predicted transmembrane domains localized in its sequence in the protein’s central portion between residues 555–574 and 643–664 (Figure 2A). The protein’s topology in parasite membrane would thus have amino and carboxyl terminal ends facing outwards, probably in contact with the host cell. Previous studies regarding *Pf*RON5 have provided contradictory results regarding the number of transmembrane domains in the sequence. Richard *et al*., predicted six transmembrane regions (TMs) for *Pf*RON5 based on a hydrophobicity profile analysis [8]. A later study used different bioinformatics tools (Phobius, Polyphobius, TMHMM2.0, ConpredII and TMpred) for constructing a consensus regarding the transmembrane domains in *Pf*RON5 [39]. Such analysis led to concluding that, as well as the different models and statistics involved in using each tool, the dissimilar results may well have been due to protozoan protein sequences having been under-represented in the training data sets of most tools in relation to other eukaryotic sequences, thereby hampering predicting transmembrane domains in these organisms [39]. Regarding *Py*RON5, two transmembrane domains were predicted by Polyphobius which had not been predicted by OCTOPUS and Phobius [49]. Interestingly, ultracentrifuge analysis supported the idea that *Py*RON5 is a protein lacking transmembrane domains, being mostly isolated in the soluble fraction compared to the fractions obtained with triton and SDS [49]. However, if the TJ model proposed by Besterio *et al*., [9] is taken into account, it may be assumed that the protein should contain at least one hydrophobic region affording passage towards host cell cytoplasm. No repeat sequences or low complexity regions were found in *Pv*RON5, contrasting with that reported for *Pv*RON2 and *Pv*RON4, where several tandem repeats have been detected between both antigens’ amino and central regions [24,25]. Repeats in malaria have been associated with evasion of the immune system regarding functionally important regions and the production of low affinity antibodies and independent T-cell activation. Interestingly, nine conserved cysteines were detected in *P. falciparum*, *P. vivax* and *P. knowlesi* RON5, distributed throughout the protein (Additional file 1), possibly being involved in conserved protein fold (Figure 2A).

Antibodies against *Pv*RON5 peptides were produced in rabbits for investigating the protein’s presence and experimental molecular weight in VCG-1 strain late schizonts (Figure 2A). The polyclonal antibodies so obtained had high reactivity (by ELISA) for the inoculated peptides compared to pre-immune sera (p < 0.01). Rabbit 32 and 60 sera recognized a single band of around 85 kDa in schizont protein extract whose intensity became reduced when the antibodies had been previously incubated with the immunized peptides (36930 and 36927) (Figure 2B). This band had a molecular weight which that was below the predicted molecular weight (133 kDa) for *Pv*RON5. Bearing studies about some rhoptry proteins (i.e., *Tg*RON5, *Tg*RON4, *Pv*RON4, *Pv*RON2, *Pf*RAP1 and *Py*RON5) in mind where proteolytic processing was identified and suggested for maturation and/or functional activation [9,24,25,49,50], anti-peptide antibodies were produced against *Pv*RON5 amino- and carboxyl-terminal extremes for identifying bands from proteolytic cleavages. Sera from rabbit 3 immunized with peptide 39276 localized towards the carboxyl-terminal extreme recognized a single ~85 kDa band, similar to sera 32 and 60 (Figure 2B), while sera from rabbit 2 inoculated with peptide 39274 located in the amino-terminal extreme recognized ~15 and ~30 kDa bands (Figure 2B), the latter band being specifically recognized, as sera previously incubated with peptide 39274 only recognized the ~15 kDa band (Figure 2B, lane 6). The ~85 and ~30 kDa specific products detected in *P. vivax* suggested that *Pv*RON5 is expressed in VCG-1 strain schizonts and undergoes proteolytic processing towards the amino terminal extreme.

Even though processing in RON5 has not been detected to date in *P. falciparum*, recent studies in *T. gondii* have shown that RON5 undergoes a minimum of two cuts, one being mediated by subtilisin 2 (SUB2) [20]. When characterizing RON5 in *P. yoelii*, the authors described a specific 87 kDa band and small ~33, ~31, and ~26 kDa fragments, suggesting physiologically processed products [48]. The *Pv*RON5 sequence was scanned for identifying possible candidate processing sites and evaluating whether they matched the consensus sequences for SUB2 (SɸXE where ɸ was a hydrophobic residue and X any amino acid) [51] and SUB1 (I/L/V/TXG/APaa (not Leu), where X was any residue and Paa tended to be a polar residue) [52]. Two probable cleavage sites were identified for SUB1, one being located in the *Pv*RON5 amino terminal region (VCGQ, residues 261–264). Such cleavage produced two fragments partly coinciding with the electrophoretic migration observed for both products recognized by WB (Figure 2B). Analysis of *Py*RON5 protein sequence (GenBank: CDZ11526.1) revealed two putative cleavage sites for SUB1 which did not coincide with the number of previously shown fragments [49]. Such marked differences in the number of fragments obtained with RON5 in *P. falciparum*, *P. vivax*, *P. knowlesi* and *T. gondii* indicated that RON5 has differing cleavage patterns and thus involves the action of different enzymes. It cannot be ruled out that differences in processing may have been due to the particular moment of the parasite’s lifecycle when the samples were taken for Western Blot analysis. However, further studies are required for evaluating putative cleavage and function regarding these proteins.

Anti-RON5 polyclonal antibodies produced in rabbits were used for determining RON5 sub-cellular localization in *P. vivax* schizonts. The images revealed that *Pv*RON5 had a dotted pattern characteristic of apical localization (Figure 2C). Including antibodies against proteins localized in the rhoptry neck, such as *Pv*RON2 and *Pv*RON4 in IFA, showed that *Pv*RON5 overlapped with the fluorescence for these two proteins, suggesting that *Pv*RON5 could be localized in the same compartment as *Pv*RON2 and *Pv*RON4 (Figure 2C) and was similar to that reported by electron microscope for *Pf*RON5 [21] and *Py*RON5 [49]. This expression and localization data agreed with antigen transcription time and coincided with the results from a previous study of the VCG-1 strain proteome where *Pv*RON5 peptides in schizont phase were identified [53].

### Determining the antigenicity of a *Pv*RON5 fragment

**Figure 3:**
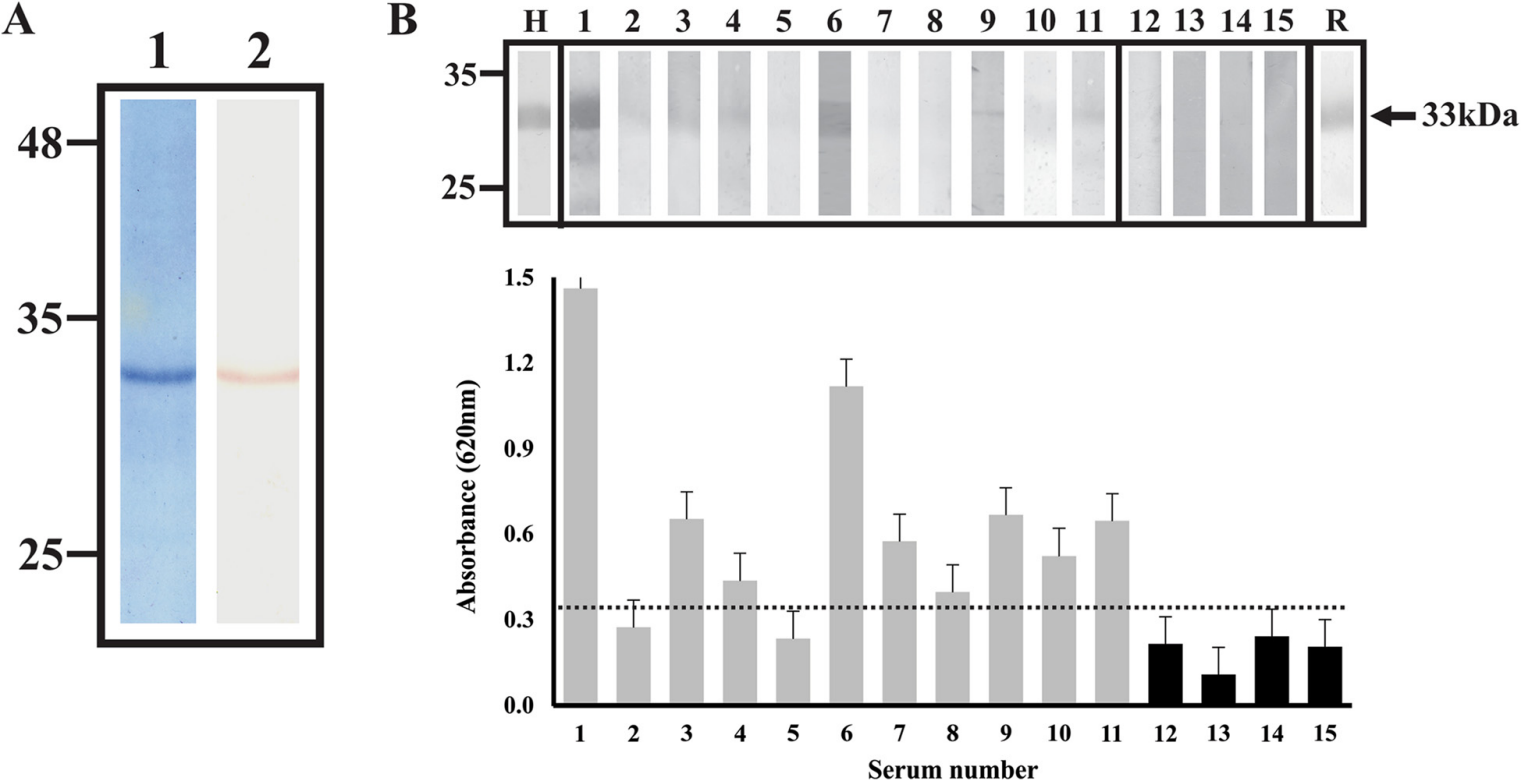
**r** ***Pv*** **RON5 expression and antigenicity studies. (A)** r*Pv*RON5 expression and purification. Lane 1, r*Pv*RON5 purified by affinity chromatography with Coomassie blue staining. A single ~33 kDa band was observed which coincided with the expected molecular weight. Lane 2, WB recognition of r*Pv*RON5 using anti-polyhistidine monoclonal antibody. **(B)** *P. vivax* patients’ sera reactivity with r*Pv*RON5. Top panel, recognition of recombinant protein by WB. H: reactivity by monoclonal anti-polyhistidine serum. Lanes 1 to 11, recognition of r*Pv*RON5 by sera from *P. vivax*-infected patients. A single ~33 kDa band can be seen. Lanes 12 to 15, sera from healthy individuals. R: Polyclonal serum 2 reactivity with r*Pv*RON5. Bottom panel, ELISA recognition of r*Pv*RON5 by sera from *P. vivax*-infected patients (grey bars) and healthy individuals (black bars).

A ~33 kDa fragment was expressed in *E. coli* and purified by affinity chromatography based on the predicted topology for *Pv*RON5 and findings regarding the function of the RON5 carboxy terminal region in *T. gondii* (Figure 3A). This recombinant protein was used for investigating (by ELISA and WB) antibodies’ natural response against *Pv*RON5 in samples from *P. vivax*-infected patients. Preliminary results showed that the sera recognized a 33 kDa band with differing reactivity, which was correlated with ELISA assay data (Figure 3B). The differences so observed in recognition of r*Pv*RON5 by sera from infected patients from different geographical regions in Colombia may have been due to the temporary acquisition of antibodies against the *Pv*RON5 carboxyl-terminal region, as has been described for the RON6 carboxyl terminus in *P. falciparum* regarding sera from patients from Papua New Guinea and Vietnam [54]. Sera from healthy individuals did not recognize r*Pv*RON5. It is worth emphasizing that reactivity was only evaluated regarding the protein’s carboxyl-terminal extreme. Future antigenicity studies could deal with evaluating the protein’s other regions.

### r*Pv*RON5 bound to samples enriched with human reticulocytes

Designing an anti-malarial vaccine has been based on a functional approach for several years now; this has involved identifying conserved regions from antigens specifically binding to host cell receptors with high affinity. An attempt was made to block regions actively involved in RBC entry and whose sequences have a significant role in invasion characterized by being much more conserved than non-critical regions [55]. This concept has led to the functional regions of most *P. falciparum* proteins participating in merozoite entry to RBC, having been identified and named HABPs. Interestingly, when HABPs were immunized in *Aotus* monkeys it was found that such conserved HABPs (cHABPs) were poorly immunogenic and thus did not induce protection against experimental challenge. A second approach was adopted which was orientated towards identifying HABP critical residues, which were then modified by replacing them with other amino acids having similar mass but different polarity and called mHABPs; many of these mHABPs were found to be immunogenic and protection-inducing [56]. The explanation for such immunological response was correlated with mHABP binding to purified molecules from the major histocompatibility complex (MHC) and its 3-D structure. This information led to suggesting that changes in cHABPs improve peptide coupling in the MHC thereby optimizing immunological presentation and thus inducing a protective immune response [57].

The lack of a continuous source of reticulocyte-enriched samples regarding *P. vivax* has hampered the identification of regions where *P. vivax* antigens bind to their target cells, using the methodology adopted concerning *P. falciparum*. Binding regions have been identified in three *P. vivax* proteins to date: *Pv*MSP1, *Pv*DBP and *Pv*RBP-1 [58-60]. Two qualitative methodologies have been adopted to make further advances in identifying these regions; they require fewer target cells for evaluating whether the *Pv*RON5 carboxyl terminal region interacts with umbilical cord samples and reticulocyte-enriched samples.

**Figure 4:**
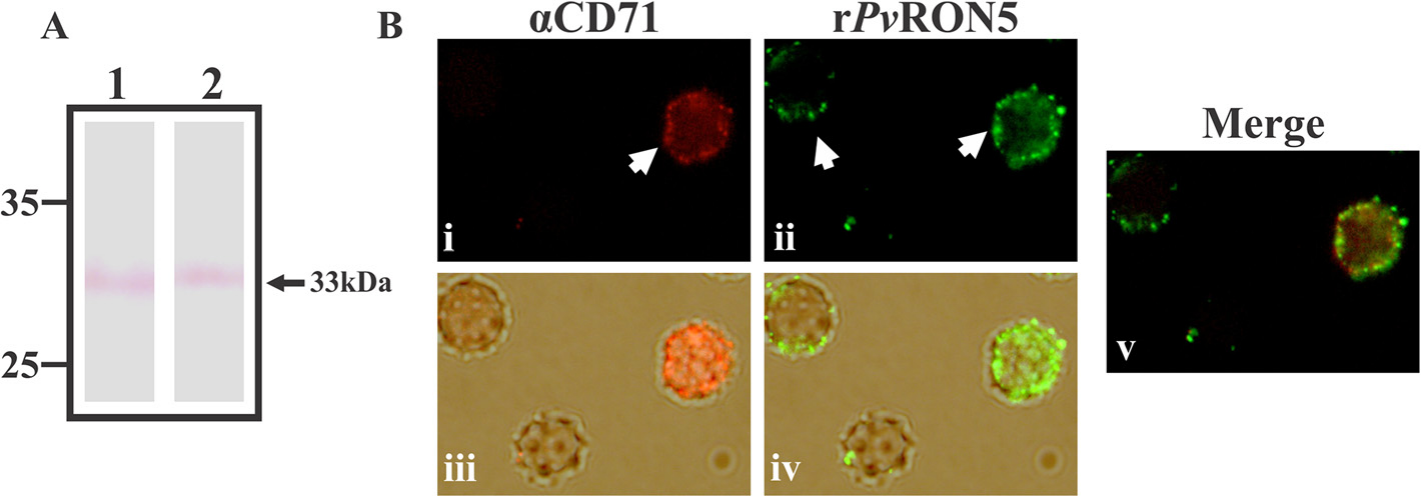
**r** ***Pv*** **RON5 binding to human reticulocytes. (A)** Immunoprecipitation and WB assays with reticulocytes (lane 1) and packed RBC (lane 2) with r*Pv*RON5. 15 μg r*Pv*RON5 was incubated with human reticulocytes or packed RBC passed through CF11 columns, followed by separation by dibutyl phthalate gradient. Cell-associated protein was eluted with 1 M NaCl in PBS and the eluted fraction was immunoprecipitated with anti-polyhistidine antibodies. The precipitate was run on an SDS-PAGE gel and revealed with anti-polyhistidine antibodies. **(B)** Indirect immunofluorescence binding studies. **(i)** reticulocyte labelling with antibodies against the transferrin receptor (CD71) which is expressed at high levels during erythroid development phases and is absent in mature cells (RBC). **(ii)** r*Pv*RON5 recognition by polyclonal antibodies directed against the recombinant bound to reticulocytes and RBC. **(iii)** The cells were seen in white light and those which were positive for CD71 (red). **(iv)** The cells were observed in white light simultaneously with r*Pv*RON5 binding (green) to cell membrane. **(v)** Merging between red fluorescence (CD71) and green (r*Pv*RON5). Greater r*Pv*RON5 preference for reticulocytes than mature RBCs was observed.

Figure 4A shows that r*Pv*RON5 bound to umbilical cord RBC and reticulocyte-enriched samples. IFA involving reticulocytes labelled with anti-CD71 antibody and polyclonal serum (rabbit 3) against r*Pv*RON5 having high reactivity for the recombinant protein as shown by WB (Figure 3, lane R), were used for determining recombinant protein preference and/or tropism for human reticulocytes or mature RBC. The results showed that r*Pv*RON5 bound to both mature RBC and reticulocytes (Figure 4B). The differences between cell-associated fluorescence measured by Image J 1.48 in RBCs compared to reticulocytes showed that r*Pv*RON5 had greater ability to bind human reticulocytes (p < 0.01) than RBCs (as expected), as reticulocytes are target cells for *P. vivax*. This pattern was similar to that reported for *Pv*MSP1 which contains reticulocyte binding HABPs, binding to erythrocytes to a lesser degree [58,61]. The foregoing could depend on the few RON5 receptors on RBC membrane compared to reticulocytes as part of RBC maturation and differentiation [62]. No fluorescence was observed in the negative controls (Additional file 2). All the above data suggested that *Pv*RON5 appears to be an adhesin, which might participate in invasion by binding to reticulocyte surface.

## Conclusions

Identifying and characterizing new antigens in *P. vivax* is an indispensable step for making advances in designing a vaccine against this parasite species. The present study identified the *pvron5* gene consisting of 8,076 bp, which was transcribed during the parasite’s intra-erythrocyte cycle and expressed at the apical pole of VCG-1 strain schizonts. The *Pv*RON5 protein contained a hydrophobic signal sequence, two transmembrane domains and was conserved between distinct strains of the parasite distributed around the world. The *Pv*RON5 carboxyl-terminal region bound to RBC, having a high preference for human reticulocytes, and was recognized by sera from *P. vivax*-infected patients. All these characteristics led to cataloguing this new antigen as an important candidate to be included in immunogenicity and protection assays in experimental models.

## Additional files

Additional file 1:
**RON5 was aligned with** ***Plasmodium*** **strains.** Red shows the cysteines which were conserved among *P. vivax*, *P. falciparum* and *P. knowlesi*; blue shows the cysteines which were only conserved between *P. vivax* and *P. knowlesi.*
Additional file 2: Figure S1. Negative controls included in r*Pv*RON5 reticulocyte binding assays. This shows the fluorescence obtained for: **a)** reticulocytes or RBC incubated only with polyclonal antibody against r*Pv*RON5 followed by FITC-coupled secondary antibodies; **b)** reticulocytes or RBC incubated with only FITC-coupled secondary antibodies; **c)** reticulocytes or RBC incubated with r*Pv*RON5 followed by incubation with FITC-coupled secondary antibody.
