# Supplementary material for: The Plasmodium vivax rhoptry neck protein 5 is expressed in the apical pole of Plasmodium vivax VCG-1 strain schizonts and binds to human reticulocytes
Source: Malar J. 2015 Mar 7;14:106. doi: 10.1186/s12936-015-0619-1 (PMC4359499; doi:10.1186/s12936-015-0619-1)
Supplement: Additional file 1: — RON5 was aligned with Plasmodium strains. Red shows the cysteines which were conserved among P. vivax, P. falciparum and P. knowlesi; blue shows the cysteines which were only conserved between P. vivax and P. knowlesi. [file 12936_2015_619_MOESM1_ESM.zip › 12936_2015_619_MOESM1_ESM.rtf]

                                                                                                                                                                                 
                      *        20         *        40         *        60         *        80         *       100         *       120         *       140         *              
PvRON5-VCG : MLKYVLLLCATLAYVPVEIESRFFENMITPKLHVGRHPIKKNLKKGKENISLDKLEKNIMKNVDSINVMFDPKDKKFVPSKSKKAHIVGGFSQNTSDPSDVERSKYEKALRFLEKMNNEMIVYSTKITRELDSQEYKTLSNFKRASALLKESLATMH :  157
PkRON5-H   : MLKYVLFFCATIAYAPLGIESRFFENIITPKLHFGRNPIKKNLKNGKKNISLDKIEKNIMKNVDSINVMFDPKDKKFVPSKSKKAHIVGGFSQNTSDPSDVERSKYEKALRFLEKMNSEMIVYSTKITRELDSQEYKTLSNFKRASALLKESLATMH :  157
PfRON5-3D7 : MLKYTLLIYIIAGYFISEISNKLFDTLLPRNVFKKPKPFKKN----EIKKGIDKDEKSIMKNVDSIDVMFEPRVKRFVPSRTRKTHVVGGLSQSIPDPGDVEKSKYEKAVRFFENIKNEMINMSSKINKQLDSQDISSLNNFKRASEVLKESLATMH :  153
             
                                                                                                                                                                                 
             160         *       180         *       200         *       220         *       240         *       260         *       280         *       300         *           
PvRON5-VCG : SLDVIKNDKTVDFTKYNLEWYAKASLKEKYETEKYIHRLMNKMFKTAGKKKKNVQKKKIDENIEQLENDLLMQRFVSENINVSKLLKEHEGKSPNYISPMHSDVCGQLGSTFLSFMFEKLYKSAMSHDLPHFKQYLPRLKQRIHQMIHKGTLILLEK :  314
PkRON5-H   : SLDMIKNDKTVDFNKYNLDWYNKASLKEKYETEKYIHRIMNKLFMKASKNKKNVQKKKIDENIEQLENDLLMQRFVSENMNVSKLLKEHEGKSPNYMAPMHSDVCGQLGSTFLSFMFEKLFKSAINHDIPHFKQYLPRLKHRIHNMVHKGTLILLEK :  314
PfRON5-3D7 : SLDIIRNDGSVDFSKYTLDWYSKANMREKYSIEKSIQKIMNKLFKKARKKKKNMKKKKIDANIEQLEMDLLVQKFITENLNASKLLKLYDDSANDYVSPMHTDVCGQLGSIIFSYMFEKAYKSSINHDISYFQKYLPRLKYRIQNMIQKGTLLLLEK :  310
             
                                                                                                                                                                                 
                320         *       340         *       360         *       380         *       400         *       420         *       440         *       460         *        
PvRON5-VCG : GLDDALHTFKLKVAELMQKKFGFVDMCSNKCIAETVNASYDLEEYQNEFKPTNTSQRRADMVKMLMYYYRDKLNNIETTADFVLIMLLYLNSATEHTEKGFIDVSSISTSDKFNLLNTTIDKRKKVK---KNKRKSFLKIAPFNFFREEPDERYGNE :  468
PkRON5-H   : GLDDALHTFKLKVAELMQKKFGFVDMCSTKCVADTVNANYDLEEYKNEFKPTNTSQRRADMVKMLMYYYRDKLNNIETTADFVLIMLLYLNSATEHTKKGFIDVSSISTTDKFNLLNTTIDKRKKVK---KNRRNSFLKIAPFNFFREEPDEKYGNE :  468
PfRON5-3D7 : GLDDSLYTFKSKISDIMEKKFGFNDMCTDKCMDETIKADYDLSEYKNEFSPSKTAQRRADLVKLLMYYYRDKIYNIETSADVVLIMLLYLNSANELSEKGYLDVSSISTDDEFNLINKTIDRSHKFNKKIKIKKKTFFKIAPFNFFREETQEKTGNE :  467
             
                                                                                                                                                                                 
                   480         *       500         *       520         *       540         *       560         *       580         *       600         *       620               
PvRON5-VCG : SIFAIDDIIKTCLLAKKSQNFSSLYETTKEVWNSIQSIYSASYGFVAGKKLKTKSFIASRIRNVGFVFNWFNYNAKASAHVNFLVHNFSPLLSVSLQLTFFISTMIEQYEASFLSNFSSTLKKIFTLGASSAHPRNYADLVSFSETDYLLRHSKADK :  625
PkRON5-H   : SIFAIDDIIKTCLLAKKSQNFSSLYETTKEVWNNIQNIYSASYGFVASRKLKTKSFVTSRIRNVGFVFNWFNYNMKPTPHVNFLVHNFSPLISVSLQLTFFISTMIEQYESSFLSNFSSTLKKIFTLGASSAHPKNYADLVSFSETDYLLRHSKTDK :  625
PfRON5-3D7 : SIFAIDDIIKTSLLAKKSQNYNSLYETTKDLWNQIQNMYSASYGFVQSKKIKTNKFVGSKIRNVGFLLRWFNYNKTPSKNINFLVNNFSPLVSISLQLVFFITTMIEQYESSFLGNFSSALKKIFTLGKSGRNPRNYNDLVNFSEVDYLLRTSKANN :  624
             
                                                                                                                                                                                 
              *       640         *       660         *       680         *       700         *       720         *       740         *       760         *       780            
PvRON5-VCG : AQRIITQTVKMLKKKFLSLPYTPTLLAQYISLFLSLWVFENERNISLENPNVTRFKKLFFLSYFVHNSGPAEKAVEIIYDRCRGKTDKIVLGCIHDYGGAKQKKLLGIINKQCKPTKIPIRKRSIRKVIKTLMSSLTDPVDILKIAVDTATRCDHFS :  782
PkRON5-H   : AQRIITQTVKMLKKKFLSLPYTPTLLAQYISLFLSLWVFENEKTISLENPNVTRFKKLFFLSYFVHNSGPAEKAVEIIYDRCRGKTDKIVLGCIHDYGGAKEKKILGIIKKKCKPTRIPIRKRSIRKVIKTLMSSLTDPVDILKIAVDTATRCDHFS :  782
PfRON5-3D7 : VQRIIMQIIRMLKKKFLSSSYTPTLLAQYMSIFLSLWVFEGENNISLQNPNISRFKKIFFLTYFVHEKGPVEKAVDIIYNKCRMKTDKIVLGCIHDYGGREKKKLLGLISRKCKPTKISIRKRSIRKILNKLMSSLNDPVDILRIAVDTATRCDHFN :  781

                                                                                                                                                                                 
                 *       800         *       820         *       840         *       860         *       880         *       900         *       920         *       940         
PvRON5-VCG : RSASMDNNKKSRNKINYDLFVKSELSFRYICADVTKKVVKKIIRDVSRLKNMSEAQELIDQSLNSVQYLKIRNYRDKESSTSIFCPFMEANDKHIRDLERKQISIFVHKNVGILNLLKGKVANVFKKSINIREGIKTDSPISIKVGMRKFNGFLFTG :  939
PkRON5-H   : RSAPMHDKKKKKNKINYDLFVKSELSFRYICADVTKKVVKKIIRDVSRLKNMSEAQEIIDQSLNSVQYLKIRNYRDKESSTSIFCPFMEANDKHIRDLERKQISIFVHKNVGILNMLKGKVANVFKKSIKIREGMKTDSPISIKVGTRKFNGFLFTG :  939
PfRON5-3D7 : RSKNIDNVKTKKNKINYEIFVKSELSIRYICADVTKNVVKKIIRDVSRLKNMREAQNVIDNGLNSVQYLKIRNYRDKESSFTILCPFMEGNDKNIRELERTQISLFIHKNIGMSRIIKGKLINIFKKTLNMREGIKSDSAISIKVGARKYNGIIFTG :  938

                                                                                                                                                                                 
                    *       960         *       980         *      1000         *      1020         *      1040         *      1060         *      1080         *      110       
PvRON5-VCG : GYQLNMDSFEQENTLHIGLSKSRKVYDGRQFVDELEILKADGVKRIEMKGIDEDNERFYVLQDKTKVPEFEYAILYPSADIIIFDGNNYVSSSALRDMGLEYERIVWAGNTVGWVAEFALGTISENPLPIFDGHAWVLLDKLSVKSILGEFLPRDVR : 1096
PkRON5-H   : GYQLNVDSFGQENTLHIGLSKSRKVYDGRQFVDELEILKPDGVKRIEMKGIDEDNERIYVLQDNTKVPEFEYAILYPSADIIIFDGNNYVSSSALRDMGLEYERIVWAGHAVGWAAEFALGTISENPLPIFDGHAWVLLDKLSVKSILGEFLPRDVR : 1096
PfRON5-3D7 : GYQLNVDNLDQ-NTLHIGLSKTRKVYDGRKYVDELEILKGDGVKNIYMKGLNEDNERIYELQNNMRVSEFDYAIQNPEANIIVFDGNNYISSYALRNMGLEHERIVWAGPSVGWTAEFALSAISDNPLPIFDGSAWVLLEKLSIRSILGKHLPSDVN : 1094
             
                                                                                  
             0         *      1120         *      1140         *      1160        
PvRON5-VCG : GSLLASTVNFIILNKEGRQILKNTTPVVSLKHATFTLSGILNFVIRAEKGKGNEIIVHTRIP : 1158
PkRON5-H   : GSLLASTVNFIILNKEGRQILKNTTPVVSLKHATFTLSGILNFVIRAEKGKGNEIIVYTRIP : 1158
PfRON5-3D7 : GNSLANTVNFVILNKDGKPILKNTTPVINLKYATFTLSGIVNFVIKAEKGIGNEIIVHTRIP : 1156
