# Supplementary figures and images for: The Plasmodium vivax rhoptry neck protein 5 is expressed in the apical pole of Plasmodium vivax VCG-1 strain schizonts and binds to human reticulocytes
Source: Malar J. 2015 Mar 7;14:106. doi: 10.1186/s12936-015-0619-1 (PMC4359499; doi:10.1186/s12936-015-0619-1)

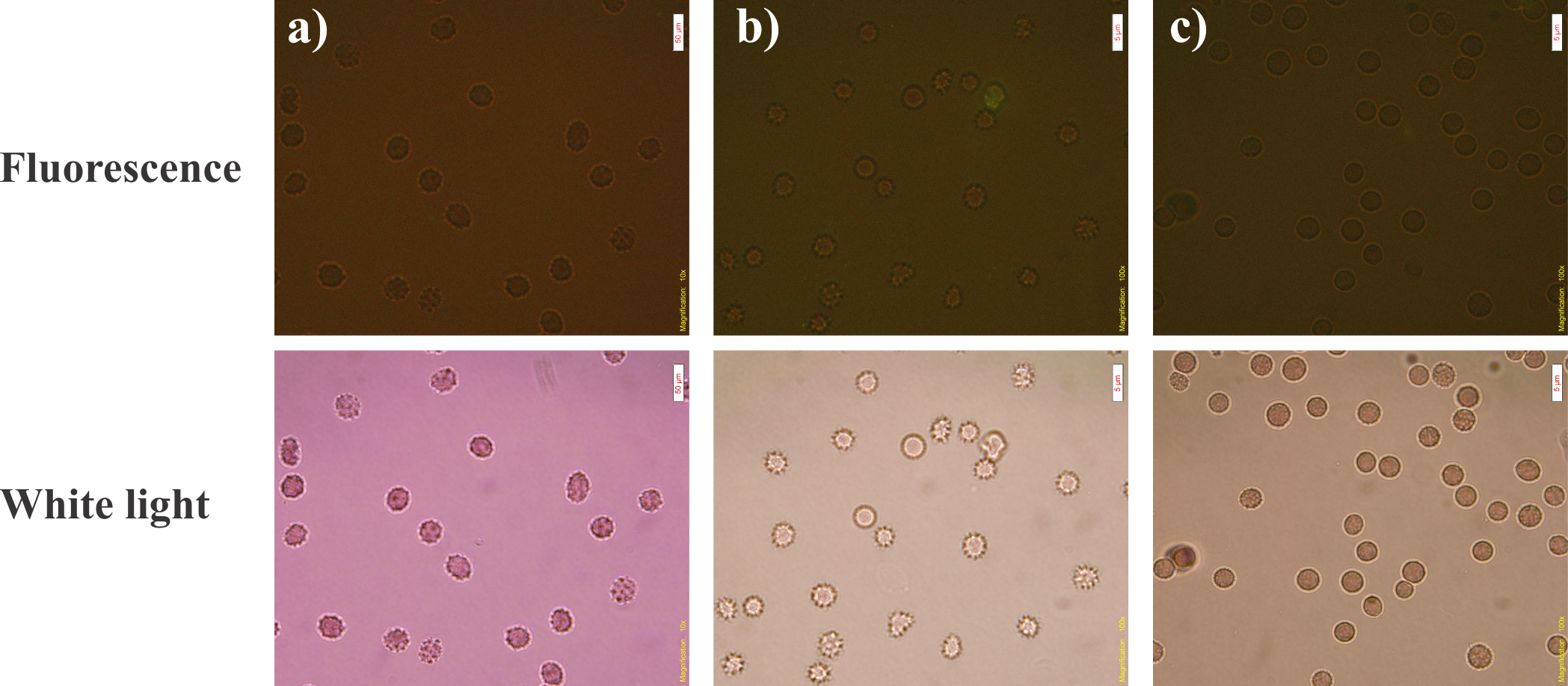

Supplement: Additional file 2: Figure S1. — Negative controls included in rPvRON5 reticulocyte binding assays. This shows the fluorescence obtained for: a) reticulocytes or RBC incubated only with polyclonal antibody against rPvRON5 followed by FITC-coupled secondary antibodies; b) reticulocytes or RBC incubated with only FITC-coupled secondary antibodies; c) reticulocytes or RBC incubated with rPvRON5 followed by incubation with FITC-coupled secondary antibody. [file 12936_2015_619_MOESM2_ESM.tiff]
